# Complement meets Tau: Exploring the effects of a novel C3‐targeting siRNA in a Tauopathy mouse model

**DOI:** 10.1002/alz70861_108945

**Published:** 2025-12-23

**Authors:** Maria‐Tzousi Papavergi, Salome Funes, Tara Barbour, Yan Li, David Eyerman, Cynthia A Lemere

**Affiliations:** ^1^ School for Mental Health and Neuroscience (MHeNs), Department of Psychiatry and Neuropsychology, Maastricht University, Maastricht, Limburg Netherlands; ^2^ Ann Romney Center for Neurologic Diseases, Brigham and Women's Hospital, Harvard Medical School, Boston, MA USA; ^3^ Apellis Pharmaceuticals, Waltham, MA USA

## Abstract

**Background:**

Complement C3 is a potential therapeutic target in AD. Tau P301S (PS19) mice have emerged as a powerful tool in studying tau pathology. Complement C3aR has been shown to be critical in mediating immune homeostasis and tau pathology in this model, while C3 deficiency mitigated neurodegeneration and neuronal loss. siRNAs enable gene silencing via RNA‐interference (RNAi). Here, we aimed to silence C3 in aged PS19 mice using a rodent active siRNA, APL‐6783, and investigated its effects on the brain and periphery.

**Method:**

At 8 months of age, male and female PS19 mice with robust tau pathology were randomly assigned to one of three treatment groups: vehicle control (PBS), low‐dose APL‐6783 (3 mg/kg), and high‐dose APL‐6783 (12 mg/kg). Mice received four subcutaneous injections, administered every two weeks over a six‐week period. One week post final dose, all mice underwent behavioral testing using the open field task, and were euthanized one week later at approximately 10 months of age. A baseline group of PS19 mice euthanized at 8 months of age was also included.

**Result:**

Behavioral testing revealed reduced anxiety‐like behavior in low‐dose‐treated mice, which spent more time in the center of the arena compared to high‐dose mice, resembling wild‐type behavior. C3 plasma levels were significantly reduced by 80% and 89% in the low‐ and high‐dose groups, respectively, compared to vehicle. Similarly, C3 protein levels in the liver, kidney, hippocampus, cortex, and cerebellum were reduced by 80‐90% in both treatment groups. Liver C3 mRNA levels decreased by 91% and 96% in the low‐ and high‐dose groups, respectively, whereas no reduction was observed in the cerebellum. C1qa mRNA levels in the cerebellum were increased approximately 6‐fold in the high‐dose group compared to vehicle. AT8 immunofluorescent staining demonstrated no significant differences in hippocampal pTau expression between groups.

**Conclusion:**

Our results indicate effective peripheral C3 protein reduction by APL‐6783, with dose‐dependent effects on behavior and complement‐related gene expression. Additional analyses are underway to further unravel the impact of C3 lowering in mice with advanced tau pathology on tau conformation, gliosis and neurodegeneration, and to better define the therapeutic potential of targeting C3 in AD.

